# Gastrointestinal helminth parasites of urban and rural foxes around Melbourne, Australia

**DOI:** 10.1016/j.ijppaw.2025.101147

**Published:** 2025-10-15

**Authors:** Bridget M. Graffeo, Ghazanfar Abbas, Charles Gauci, Kabir Brar, Leonardo Brustenga, Tharaka Liyanage, Megan Fisher, Jessica Haining, Jasmin Hufschmid, Ian Beveridge, Abdul Jabbar

**Affiliations:** a Department of Veterinary Biosciences, Melbourne Veterinary School, The University of Melbourne, Werribee, Victoria, Australia; b Department of Veterinary Medicine, University of Perugia, Perugia, Italy

**Keywords:** Australia, Urban foxes, Cestodes, Nematodes, Gastrointestinal parasites

## Abstract

The red fox (*Vulpes vulpes*) is an introduced species to Australia whose population and spatial distribution have grown irreversibly. Due to their opportunistic feeding habits, extensive populations of foxes now inhabit urban and rural environments, where they coexist with humans and domesticated animals. The proximity of these predators presents public and animal health concerns as they harbour diseases that can cross between species. Accordingly, monitoring potential disease risk and prevalence in urban foxes is warranted. This study investigated the occurrence of gastrointestinal parasites in urban and rural foxes around Melbourne, Victoria, Australia. The gastrointestinal tracts of 51 opportunistically collected foxes were thoroughly examined to collect adult helminth (i.e., nematode and cestode) parasites from the stomach as well as from the small and large intestines. The results showed that 92.2 % of foxes were infected with at least one gastrointestinal helminth parasite. Based on a morphological identification of worms, the detected nematode parasites were *Toxocara canis* (66.7 %) and *Uncinaria stenocephala* (56.9 %)*,* while the identified tapeworms included *Dipylidium caninum* (39.2 %), *Taenia* spp. (11.8 %) and *Spirometra mansoni* (5.9 %). Single cases of *Trichuris vulpis* and *Physalopetra* sp. infections were detected. This study highlights a human and domestic animal health risk, as a crossover of parasitic infections is possible in areas where these parasites coexist.

## Highlights

• Urban foxes can pose public and animal health risks.
• Examination of 51 fox guts showed that 92 % were infected with at least one helminth parasite.
• Common parasites detected were *Toxocara canis* (67 %), *Uncinaria stenocephala* (57 %) and *Dipylidium caninum* (39 %).
• Parasite crossover between foxes, humans, and domestic animals poses a health risk in shared habitats.

## Graphical abstract

**Figure:**
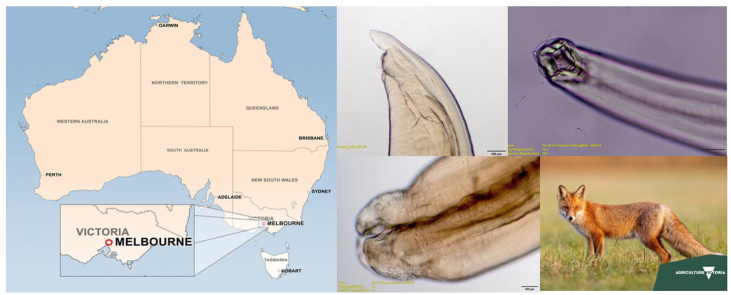

## 1 Introduction

The red fox (*Vulpes vulpes*) has a significant impact on urban, peri-urban and rural ecosystems, contributing to both ecological and public health concerns. Remarkably, the adaptability of foxes to human-modified landscapes has enabled their widespread colonisation of Australian cities, including Melbourne, where foxes thrive by exploiting anthropogenic resources such as food, water and shelter (Marks and Bloomfield, 1999; Bateman and Fleming, 2012; Campbell et al., 2020). High reproductive capacity, dietary flexibility and behavioural plasticity (McIntosh, 1963; Coman, 1973a) allow foxes to persist at elevated densities in close proximity to people and domestic animals (Saunders et al., 1995). While they influence urban ecosystems by feeding on small vertebrate populations (Harris and Rayner, 1986; Soulsbury and White, 2015), foxes also act as reservoirs for a range of zoonotic parasites, posing significant and often under-recognised risks to public health (Plumer et al., 2014; Hobbs et al., 2025).

Urban foxes in Australia are known carriers of helminths such as *Dipylidium caninum*, *Echinococcus granulosus* and *Toxocara canis*, all of which can cause disease in humans, including visceral and ocular larva migrans, and life-threatening hydatid disease (Jenkins et al., 2005; Thompson, 2017; Karamon et al., 2018). The shedding of infective parasite stages in animal faeces, particularly in public spaces (e.g., parks), contributes to environmental contamination and increases the risk of spillover to humans and pets (Carden et al., 2003; Massetti et al., 2022). This risk is exacerbated by the survival of helminth eggs which can persist in the environment for prolonged periods (Macpherson, 2013).

Although the zoonotic importance of these parasites is well-established, fox control methods commonly used in rural areas, such as baiting, den fumigation, shooting and trapping, are impractical in urban environments due to risks to non-target species and public safety (Jenkins and Craig, 1992). As a result, urban fox populations remain largely unmanaged and surveillance data on parasite prevalence are lacking. While several studies have investigated helminths in rural foxes and wild dogs in other parts of Australia (Coman, 1972; Jenkins and Morris, 2003; Harriott et al., 2019), contemporary data from urban centres such as Melbourne are scarce.

This study addresses this critical knowledge gap by investigating the prevalence and diversity of gastrointestinal (GI) helminths in red foxes from metropolitan Melbourne. The findings aim to inform zoonotic disease risk assessments, support One Health public health strategies, and guide evidence-based policy and wildlife management initiatives.

## 2 Materials and methods

### 2.1 Study context and population

Red foxes were introduced to Australia in the 1850s for recreational hunting and have since established populations across the continent (Saunders et al., 2010). Only 20 years after their introduction, foxes were declared as a pest species in Victoria (https://agriculture.vic.gov.au/biosecurity/pest-animals/established-pest-animal-species/red-fox). Approximately 1.7 million foxes currently occupy more than 80 % of mainland Australia, excluding only dense tropical rainforests and some arid zones (Dielenberg et al., 2022). In comparison to rural foxes, urban and peri-urban foxes exhibit more confident behaviours, likely due to a reduced predator-prey overlap. This reduction results from limited interactions with an apex predator, in this case the dingo (*Canis lupus dingo*), and from predominantly nocturnal activity patterns that minimise encounters with domestic dogs and humans (Campbell et al., 2020; Gil-Fernández et al., 2020).

### 2.2 Sample collection

**Fig. 1:**
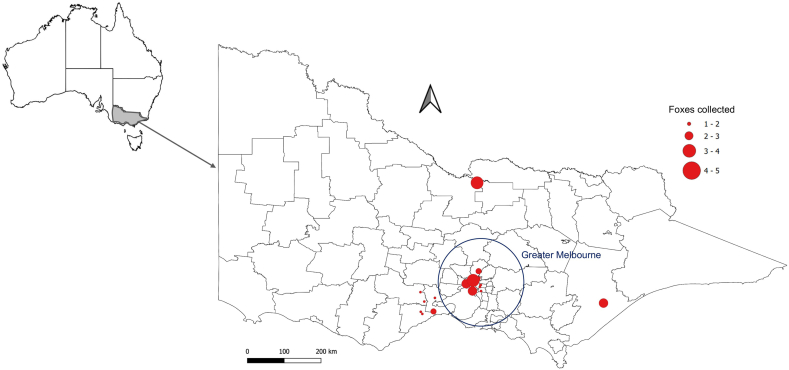
Map of Australia with an inset showing the state of Victoria, highlighting the fox collection sites. Red circles represent collection locations, with circle size proportional to the number of foxes collected at each site.

A total of 51 red foxes were collected for this study, including 31 from the greater Melbourne area and 20 from rural regions of Victoria (Fig. 1). Samples were collected opportunistically, with all animals culled by professional hunters as part of government-approved vertebrate pest control programs. The gastrointestinal tract (GIT) of each fox was removed after the abdomen was opened and stored at −18 °C until examination. Sex, estimated age, body weight and body condition score (BCS) were recorded for each individual based on visual inspection of the carcass, by palpation and comparison of body weight (measured on an electronic scale) with reference values for healthy foxes. BCS was attributed to each animal by the same person to avoid inter-observer variability and was given a scoring from 1 to 5 based on fat deposit indices previously described by Cavallini (1996). BCS was based both on external appearance of the animal and on the presence of perivisceral fat: BCS 1 represented severely malnourished animals with visible bones and no fat deposits; BCS 2 for emaciated animals with no fat deposits; BCS 3 for animals with subcutaneous fat deposits over the sternum and little perivisceral fat deposits scattered throughout the omentum; BCS 4 for animals with discontinuous subcutaneous fat, perirenal fat and perivisceral fat not completely encasing the intestines; and BCS 5 for animals with continuous subcutaneous fat deposits, perirenal and perivisceral fat that encased the intestine allowing for its removal in a single block. Individual animals were assigned either a juvenile or adult age class based on incisor tooth eruption and wear (Harris, 2009) and body mass. Juveniles were defined as foxes weighing under 3.5 kg that still had deciduous teeth. Foxes under or above 3.5 kg with permanent or worn teeth were considered adults. This integrative method helped reduce classification errors due to dental damage and also accommodated variations in body condition related to nutrition.

Prior to dissection, frozen GITs were defrosted at room temperature for 24 h. The omentum was removed to linearise the GIT tract, which was then divided into the stomach, small intestine and large intestine. Each section was individually examined for gross abnormalities and parasite content. The stomach was incised along the greater curvature, and its contents were washed into a container. Both the small and large intestinal contents were separately flushed three times with water to collect parasites.

The contents of each GIT segment were allowed to settle in separate plastic containers filled with water. Sedimentation was achieved by repeated decanting and refilling until only particulate matter remained. The timing of each sedimentation varied depending upon the contents in each sample. The residual sediment was transferred to sample containers and preserved in 70 % ethanol. In cases where cestodes were observed, specimens were suspended in saline for 1 h prior to fixation. Each sample container was labelled with the corresponding fox ID and GIT segment. Contents were later transferred to Petri dishes and examined under a dissecting microscope and all helminths were removed and counted using the standard parasitological technique known as total worm counts (TWC).

### 2.3 Helminth examination

Helminths were identified to genus or species level based on morphological characteristics, following standard taxonomic keys. Smaller specimens were mounted whole in lactophenol on glass slides. For larger helminths, only the anterior and posterior ends were mounted for identification. Samples were allowed to clear in lactophenol for 24 h.

Helminths were initially identified following the standard texts of Taylor et al. (2015) and Bowman (2021a). Subsequent identification utilised more specialised literature. *Toxocara canis* was identified following Warren (1971), *Uncinaria* following Lichtenfels (1980) and *Trichuris* following Anderson and Bain (1982). The larval physalopterid was identified following Bain et al. (2014).

Cestodes were identified macroscopically by evaluating body length, segment morphology, and the location of the genital pore, in accordance with Jacobs et al. (2016). Terminology for species of *Spirometra* species follows Kuchta et al. (2024). *Taenia* species were further differentiated by measuring rostellar hook lengths following the methods previously described by Beveridge and Gregory (1976). However, species-level identification was not possible in some cases due to specimen damage or an absence of the scolex.

### 2.4 Statistical analysis

Descriptive statistics were undertaken using R version 4.3.1 (R Core Team, 2023) to analyse the parasite prevalence (both nematode and cestodes), their burdens and individual parasite prevalences. The data were further grouped by age, sex, BCS and collection location to estimate the prevalence and mean intensity within each group. Prevalence was presented as proportions with 95 % confidence intervals (CI). Density plots were generated to visualise the distribution of nematode intensity across host categories (age, sex, body condition score and location). These plots present smoothed probability density estimates of the observed parasite counts, without transformation of the raw data. Associations between parasite (including both nematodes and cestodes) prevalence and host intrinsic (sex, age and BCS) and extrinsic (location) factors were assessed using generalised linear models (GLM). Considering the potential bias associated with the small sample size (*n* = 51), Firth's penalized logistic regression, a common approach in wildlife studies, was used for multivariable analysis.

For parasite intensity (nematode count per infected individual), a negative binomial regression model was used, considering the overdispersion in the data. The same fixed effects (age, sex, BCS and location) were tested for the parasitic burdens. Model fit was evaluated using residual diagnostics, dispersion statistics and likelihood ratio tests. Regression outputs were reported as odds ratios (OR) for logistic models and incidence rate ratios (IRR) for count models with corresponding 95 % CIs and p-values. A p-value <0.05 was considered statistically significant.

## 3 Results

### 3.1 Prevalence of GI helminths

Of the 51 fox GITs examined, 92.2 % (*n* = 47; 95 % CI: 81.5 to 96.9) were infected with at least one species of helminth. Most foxes were infected with nematodes (82.4 %; 42/51, 69.7 to 90.4), whereas half (51 %; 26/51, 37.7 to 64.1) had cestodes and 41.2 % (21/51, 28.8 to 54.8) had both cestodes and nematodes.

**Fig. 2:**
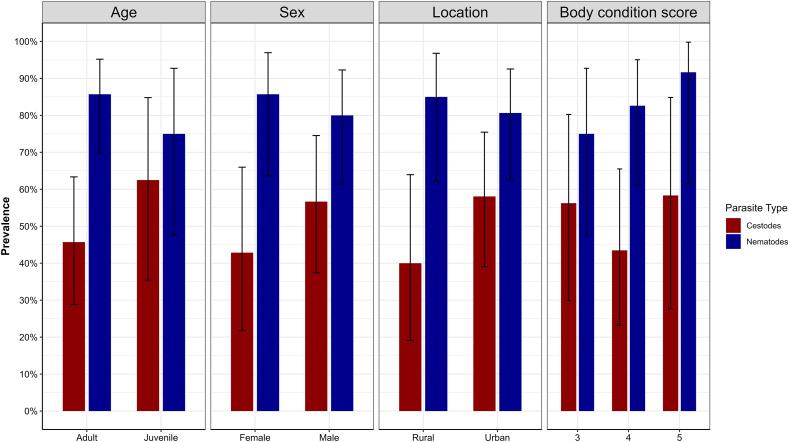
Prevalence of nematode and cestode parasites in red foxes collected across Victoria. The prevalence was estimated based on the presence of adult worms. Error bars represent 95 % confidence intervals.

The observed prevalence of nematodes was 85.7 % (95 % CI: 69.7 to 95.2) in adults, 85.7 % (63.7–97.0) in females, and 85 % (62.1–96.8) in rural foxes (Fig. 2). Across the BCS, prevalence ranged from 75 % (95 % CI: 47.6 to 92.7) in foxes with BCS 3–91.7 % (95 % CI: 61.5 to 99.8) in foxes with BCS 5 (Fig. 2). For cestodes, prevalence was 62.5 % (95 % CI: 38.6 to 81.5) in juveniles, 56.7 % (39.2–72.6) males, and 58.1 % (40.8–73.6) in urban foxes, with relatively consistent levels (43.5 %–58.3 %) across BCS categories (Fig. 2). No statistically significant associations were detected between parasite prevalence and intrinsic (age, sex and BCS) or extrinsic (location) factors based on Firth's penalized logistic regression model (Appendix A: Supplementary Table 1).

**Fig. 3:**
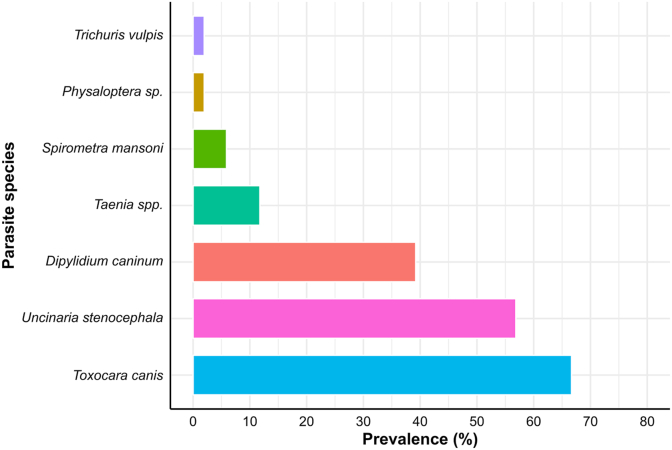
Prevalence of individual gastrointestinal parasites in red foxes. The prevalence was estimated based on the presence of adult worms.

Seven helminth taxa were identified in all the samples across Victoria (Fig. 3). The most commonly detected nematode species were *Toxocara canis* (66.7 %; 34/51) and *Uncinaria stenocephala* (56.9 %; 29/51). Cestode parasites detected were *Dipylidium caninum* (39.2 %; 20/51), *Taenia* spp. (11.8 %; 6/51) and *Spirometra mansoni* (5.9 %; 3/51) (Fig. 3). Among the six foxes infected with *Taenia* spp., two specimens were successfully identified as *Taenia serialis* by the presence of rostellar hooks. The remaining specimens had a similar proglottis morphology (Arundel, 1972). Additionally, single cases of two nematodes, *Trichuris vulpis* (1.96 %; 1/51) and a larval *Physaloptera* sp. (1.96 %; 1/51) (could not be identified to species), were detected in the large intestine of two separate foxes (Fig. 3).

### 3.2 Prevalence variations among helminth species, and intrinsic and extrinsic factors

Differences in helminth species composition were observed between foxes collected from urban versus rural areas (Table 1). The observed prevalence of *Toxocara canis* was higher in foxes originating from urban (71 %) than rural (60 %) areas. Similarly, the prevalence of *U. stenocephala* was higher in rural (80 %) than urban foxes (41.9 %), whereas *D. caninum* was more common in urban (54.8 %) than rural foxes (15 %). *Trichuris vulpis*, *Physaloptera* sp., and *S. mansoni* were only recorded in rural foxes (Table 1). *Toxocara canis* was the most prevalent parasite in both adult (65.7 %, 23/35) and juvenile (68.8 %, 11/16) foxes. High prevalences were also observed for *U. stenocephala* in adults (60 %; 21/35) and juveniles (50 %; 8/16). The prevalences of *T. canis* (73.3 %) and *D. caninum* (43.3 %) were higher in males while that of *U. stenocephala* (66.7 %) was higher in females. *Trichuris vulpis* was only detected in female adult foxes collected from rural areas (4.8 %).

**Table 1:** Prevalence (%) of individual helminth species in red foxes (*Vulpes vulpes*) across age, sex, body condition score (BCS) and location groups in Victoria, Australia.

	Category (total number of samples)	Toxocara canis (number of positive samples)	Uncinaria stenocephala	Dipylidium caninum	Taenia spp.	Spirometra mansoi	Physaloptera sp.	Trichuris vulpis
Age	Juvenile (16)	68.8 (11)	50 (8)	43.8 (7)	18.8 (3)	0 (0)	0 (0)	0 (0)
	Adult (35)	65.7 (23)	60 (21)	37.1 (13)	8.6 (3)	8.6 (3)	2.9 (1)	2.9 (1)
Sex	Male (30)	73.3 (22)	50 (15)	43.3 (13)	13.3 (4)	10 (3)	3.3 (1)	0 (0)
	Female (21)	57.1 (12)	66.7 (14)	33.3 (7)	9.5 (2)	0 (0)	0 (0)	4.8 (1)
BCS	BCS 3 (16)	68.8 (11)	50 (8)	43.8 (7)	12.5 (2)	0 (0)	6.2 (1)	0 (0)
	BCS 4 (23)	60.9 (14)	60.9 (14)	39.1 (9)	8.7 (2)	4.3 (1)	0 (0)	4.3 (1)
	BCS 5 (12)	75 (9)	58.3 (7)	33.3 (4)	16.7 (2)	16.7 (2)	0 (0)	0 (0)
Location	Urban (31)	71 (22)	41.9 (13)	54.8 (17)	3.2 (1)	0 (0)	0 (0)	0 (0)
	Rural (20)	60 (12)	80 (16)	15 (3)	25 (5)	15 (3)	5 (1)	5 (1)

### 3.3 Parasite intensity

**Fig. 4:**
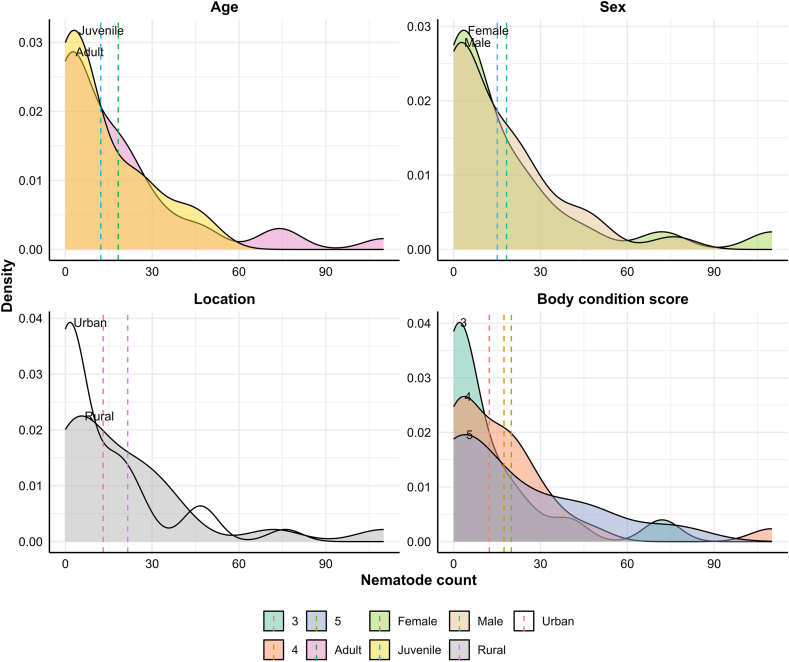
Density plots showing the distribution of parasite intensity (i.e., total worm count) in red foxes, categorised by age, sex, location and body condition score (3–5). Dotted vertical lines indicate the mean parasite intensity for each category, with different colours representing different groups within each category.

For cestodes, only their presence or absence was noted, as their counting was not possible due to the unavailability of intact worms in most of the samples. In contrast, mean TWCs were performed for nematodes found. The mean TWC and standard deviation for nematodes per fox were 16.4 ± 22.7, with individual burdens ranging from 0 to 110. Foxes from rural regions exhibited a higher mean TWC (21.6 ± 27.6), with a broader distribution, suggesting higher and more variable parasite burdens (Fig. 4) than those from the Greater Melbourne area (13.1 ± 18.6) which showed a pronounced peak at very low TWC with relatively fewer higher burdens (Fig. 4). The adult and juvenile foxes had parasitic burdens of 18.3 ± 25.3 and 12.3 ± 15.3, respectively. Both sexes had similar density curves, skewed toward lower worm counts. However, females showed a slightly more peaked distribution around low values with mean TWC slightly higher (18.3 ± 27.8) than males (Fig. 4). Lower BCS groups (e.g., BCS 3) showed higher densities at low TWCs (12.3 ± 19.6). In contrast, BCS 5 had higher densities towards TWCs (19.9 ± 24.8).

Nematode counts showed overdispersion and were best captured by a multivariable negative binomial model. After adjusting for sex, age and BCS, foxes in urban areas had significantly lower nematode counts (p value = 0.034), while BCS showed a non-significant positive trend (p value = 0.076). Other variables were not significant (Appendix A: Supplementary Table 2).

### 3.4 Mixed infections and parasite distribution

Mixed-species infections were common among helminth-positive foxes. Co-infection with two parasite species was the most frequently observed (*n* = 24), followed by single-species infections (*n* = 12), triple infections (*n* = 10), and one case of a quadruple infection. Helminths were predominantly recovered from the small intestine. However, *T. vulpis* and *Physaloptera* sp. were exclusively isolated from the large intestine.

### 3.5 Dietary observations

Analysis of GIT contents revealed various macroscopically identifiable dietary items. The most frequently observed components were insects (37.3 %), bird remains/feathers (33.3 %) and plant material (33.3 %). Less commonly detected items included berries (11.8 %) and mammalian fur (11.8 %).

## 4 Discussion

This study provides the most up-to-date evidence on GI helminths in urban and rural red foxes from Victoria, Australia. Seven helminth species were identified, several of zoonotic concern, with observed prevalence patterns varying by host age, sex, BCS and location. Notably, this is the first report of *Physaloptera* sp. in Australian foxes. Although this nematode genus poses limited zoonotic risk, some species can cause significant gastric diseases in domestic animals (e.g., dogs and cats), warranting further surveillance.

We found that the overall prevalence of GI helminths (92.2 %) was substantially higher than previously reported in Australian foxes, exceeding the rates reported by Ryan (1976; 80.6 %, *n* = 930), Coman (1973b; 71 %, *n* = 1320) and Dybing et al. (2013; 58.0 %, *n* = 147) in various Australian states, including New South Wales, Victoria and southwest Western Australia, respectively. This discrepancy likely reflects both temporal and ecological shifts. Increased fox densities, changes in diet and greater access to intermediate or paratenic hosts may have resulted in higher transmission dynamics over time. Previously, a study by Gasser et al. (1994) carried out in north-eastern Victoria demonstrated that the incidence of parasites can double within only a few years when monitored over time in the same area. Furthermore, climatic factors can also contribute to a higher prevalence of parasites. For example, Massetti et al. (2022) reported higher soil contamination with helminth eggs in temperate zones such as Melbourne compared with subtropical or Mediterranean areas such as Sydney or Perth.

Among the helminths identified in this study, the prevalences of *T. canis* (66.7 %) and *U. stenocephala* (56.9 %) were substantially higher than those reported previously in foxes in Australia (i.e., *T. canis*: 14.9 %–42.3 %; *U. stenocephala*: 18.2 %–30.6 %) (Coman, 1973b; Ryan, 1976; Dybing et al., 2013). These higher prevalences of *T. canis* and *U. stenocephala* were more aligned with those reported in the UK (55.9 % and 68.0 %, respectively) (Richards et al., 1995), suggesting similar ecological and demographic drivers, including high greenspace density, diets, overlapping domestic animal-human-fox habitats (Gecchele et al., 2020) and fox access to intermediate or paratenic hosts (Mackenstedt et al., 2015). The high prevalence of *T. canis* is likely underpinned by multiple factors, including the ingestion of paratenic hosts such as birds and rodents (Macpherson, 2013), high fox and dog densities promoting faecal contamination of the environment and the extreme resilience of *T. canis* eggs. In this study, diet analysis revealed frequent ingestion of avian material, supporting this hypothesis. Previously, the main components of the diet of Victorian foxes included rabbits, sheep carrion and mice (McIntosh, 1963; Coman, 1973a). However, it can be expected that foxes living within larger cities would shift from a rabbit/sheep-dominated diet to a more varied diet of smaller mammals, birds and human food waste. Herein, the increased prevalence of *U. stenocephala* could be attributed to the parasite's adaptation to cooler climates (Bowman et al., 2021b), consistent with southeastern Australia. In contrast, domestic dogs in Australia show much lower prevalence of helminths (Moore and O'Callaghan, 1985; Palmer et al., 2008; Gillespie and Bradbury, 2017), largely attributable to widespread use of anthelmintics and reduced opportunities for exposure to infected faeces or offal. These differences underscore the limitation of extrapolating zoonotic risk from wildlife reservoirs to domestic pets, particularly in peri-urban or rural environments.

In this study, *D. caninum* was detected in 39.2 % of foxes, a far higher level than previous reports for both foxes (Ryan, 1976; Coman, 1973b) and domestic animals (Gregory and Munday, 1976; Gillespie and Bradbury, 2017; Roeber et al., 2024). Urban foxes exhibited higher prevalences (54.8 %; 17/31), possibly due to increased flea burdens associated with dense populations and more frequent interspecies contact. In contrast, *Taenia* spp. (25 %; 5/20) and *S. mansoni* (15 %; 3/20) were more prevalent in rural foxes, reflecting their reliance on transmission through livestock/rabbits (for *Taenia* spp.) and amphibians or aquatic intermediate hosts (for *S. mansoni*) (Bennett et al., 2014). These patterns highlight how urbanisation influences parasite species composition and transmission pathways.

Notably, this study did not detect *Echinococcus granulosus*, a major zoonotic cestode of public health concern that has previously been detected in studies using similar methodologies (Harriott et al., 2019; Jenkins and Morris, 2003). While Jenkins (2006) demonstrated that foxes can act as definitive hosts in southeastern Australia, particularly in remote or inaccessible landscapes, our findings suggest that urban foxes in accessible areas of Victoria currently play a limited role in its transmission. Future studies may include mucosal scrapings to best screen for this cestode (Rojas et al., 2024).

A single fox was found positive for a spirurid nematode, *Physaloptera* sp. To our knowledge, this represents the first report of this nematode in Australian foxes. Although of limited zoonotic relevance, *Physaloptera* spp. can cause significant gastric pathology in domestic animals (Soderman and Harkin, 2020). The only known species to occur in dogs or cats in Australia is *P. praeputialis*, which occurs in the stomach and has previously been reported in the Northern Territory and Western Australia (O'Callaghan and Beveridge, 1996; Adams, 2003). The present finding of a larval stage in the large intestine of a Victorian fox is likely consistent with the *Physaloptera* sp. nematode as a pseudoparasite. Foxes coinhabiting with either of the black rat (*Rattus rattus*) or the bush rat (*Rattus fuscipes*), which can host *P. murisbrasiliensis* or *P. banfieldi,* respectively, may harbour a degenerating larva passing through the gut following ingestion of an intermediate host with a 3rd stage larva (presumably a beetle) (Smales, 2006 and unpublished data). The presence of intermediate or paratenic hosts such as insects (e.g., crickets, cockroaches, beetles) or mammals (e.g., rodents) (Campbell and Graham, 1999) in rural habitats may support this interpretation.

The prevalence of helminths did not differ significantly between male and female foxes, concurring with previous findings (Vervaeke et al., 2005; Tylkowska et al., 2021) and likely reflecting similar dietary habits and behavioural patterns. Adults, however, had a higher nematode burden than juveniles, contrary to the common assumption that young animals bear higher parasite burdens (Palmer et al., 2007; Harriott et al., 2019; Rostami et al., 2020). Gemmell (1959) proposed that dietary factors outweigh age in determining parasite loads and greater exposure to intermediate hosts. Recently, Otranto and Deplazes (2019) suggested that immunity acquisition may also play a role in the susceptibility of foxes to parasites.

Rural foxes harboured more diverse helminth communities (seven species versus four in urban foxes) and significantly higher nematode burdens. Three helminths, including *S. mansoni*, *T. vulpis* and *Physaloptera* sp. were exclusively found in rural foxes. These findings reflect the greater biodiversity and range of intermediate hosts in rural habitats (Reshamwala et al., 2018). Nevertheless, urban foxes carried parasite burdens, highlighting their role as reservoirs of zoonotic infection in human-populated environments. There is a paucity of information on the relationships between helminth life cycles and the interaction with humans, wildlife and domesticated pets which warrant further investigation (Mackenstedt et al., 2015). This study highlights the need for improved surveillance of fox populations around urban centres in order to develop appropriate management strategies where fox-human-pet interactions are frequent.

Effective management of fox populations remains challenging in Australia. Traditional methods such as sodium fluoroacetate (1080) baiting have limited utility in urban settings due to risks to domestic pets and rapid population replacement via immigration of foxes (Harding et al., 2001; Gil-Fernández et al., 2020). Experiences from European rabies control programs suggest that non-lethal, goal-oriented interventions, such as oral vaccination (for *E. multilocularis*) or targeted anthelmintic baiting, may be more sustainable (Pastoret and Brochier, 1999; Hegglin et al., 2003). However, the growing problem of anthelmintic resistance in species such as *Ancylostoma caninum* (Kopp et al., 2007; Kitchen et al., 2019; Abdullah et al., 2025) necessitates a cautious and evidence-based approach to wildlife treatments. Future research should explore innovative, non-lethal strategies that balance parasite control with ecological sustainability and minimise anthelmintic resistance selection pressures.

The findings of the present study underscore the public and veterinary health implications of fox-borne helminths in both urban and rural contexts. The high prevalence of zoonotic species, particularly *T. canis*, *U. stenocephala* and *D. caninum*, presents a potential risk of transmission to humans and companion animals in shared environments such as parks and suburban gardens (Dunsmore et al., 1984). Public awareness of these risks remains low (Massetti et al., 2023; Owada et al., 2023), highlighting the need for targeted education campaigns on parasite transmission pathways, environmental hygiene and safe practices such as avoiding feeding raw offal to pets (Jenkins and Morris, 2003).

This study was limited by a modest sample size and temporal variability in collection, which may have influenced prevalence estimates. Future studies should employ larger, temporally consistent sampling and integrate spatial tracking to better characterise transmission hotspots and inform targeted interventions.

## 5 Conclusion

In conclusion, the high burden and zoonotic potential of GI helminths in urban and rural foxes highlight the need for updated surveillance and integrative management strategies. Outdated data for biosecurity frameworks risk underestimating transmission and delaying control measures. A One Health approach, i.e., linking human, animal, and environmental health, is essential to guide public education, evidence-based veterinary practices and sustainable, non-lethal control options. Future work should prioritise long-term monitoring and spatial mapping to identify transmission hotspots and support targeted interventions that protect both public and animal health.

## CRediT authorship contribution statement

**Bridget M. Graffeo:** Writing – original draft, Visualization, Methodology, Investigation, Data curation. **Ghazanfar Abbas:** Writing – review & editing, Visualization, Formal analysis. **Charles Gauci:** Writing – review & editing, Methodology, Data curation. **Kabir Brar:** Writing – review & editing, Data curation. **Leonardo Brustenga:** Writing – review & editing, Data curation. **Tharaka Liyanage:** Writing – review & editing, Data curation. **Megan Fisher:** Writing – review & editing, Data curation. **Jessica Haining:** Writing – review & editing, Data curation. **Jasmin Hufschmid:** Writing – review & editing. **Ian Beveridge:** Writing – review & editing, Methodology, Investigation, Data curation. **Abdul Jabbar:** Writing – review & editing, Writing – original draft, Visualization, Validation, Supervision, Resources, Methodology, Investigation, Funding acquisition, Data curation, Conceptualization.

## Conflict of interest statement

The authors declare that they have no known competing financial interests or personal relationships that could have appeared to influence the work reported in this paper.

## Data Availability

All data related to this manuscript are included herein.
