# Supplementary material for: Gastrointestinal helminth parasites of urban and rural foxes around Melbourne, Australia
Source: Int J Parasitol Parasites Wildl. 2025 Oct 15;28:101147. doi: 10.1016/j.ijppaw.2025.101147 (PMC12557487; doi:10.1016/j.ijppaw.2025.101147)
Supplement: Multimedia component 1 [file mmc1.docx]

Supplementary Table 1

Regression coefficients and their standard errors (SE) from a Penalized logistic regression model (Firth method) of factors associated with parasite presence in foxes. A *p*-value < 0.05 was considered statistically significant.

| Variable | Coefficient (β) | SE | 95% CI | Chi-square | p-value |
| --- | --- | --- | --- | --- | --- |
| Intercept | -0.218 | 2.376 | -6.18 – 4.96 | 0.007 | 0.935 |
| Sex (Male) | 0.295 | 0.868 | -1.66 – 2.24 | 0.098 | 0.754 |
| Age (Adult) | 0.363 | 0.936 | -1.83 – 2.43 | 0.118 | 0.731 |
| Body condition score | 0.577 | 0.663 | -0.77 – 2.41 | 0.634 | 0.426 |
| Location (Urban) | -0.475 | 0.935 | -2.86 – 1.43 | 0.226 | 0.634 |

None of the tested variables were significantly associated with presence of parasites.

Supplementary Table 2

Regression coefficients and their standard errors (SE) from a negative binomial regression model of factors associated with nematode counts in foxes. A *p*-value < 0.05 was considered statistically significant.

| Variable | Estimate | SE | z value | p-value |
| --- | --- | --- | --- | --- |
| Intercept | 0.993 | 1.128 | 0.880 | 0.379 |
| Sex (Male) | 0.102 | 0.423 | 0.242 | 0.809 |
| Age (Adult) | 0.220 | 0.471 | 0.468 | 0.640 |
| Body condition score (5) | 0.520 | 0.293 | 1.773 | 0.076 |
| Location (Urban) | -0.897 | 0.423 | -2.120 | 0.034 * |

*Foxes in urban areas had significantly lower nematode counts compared to rural foxes.
